# Grazing intensity enhances spatial aggregation of dominant species in a desert steppe

**DOI:** 10.1002/ece3.5197

**Published:** 2019-04-26

**Authors:** Shijie Lv, Baolong Yan, Zhongwu Wang, Guodong Han, Sarula Kang

**Affiliations:** ^1^ College of Grassland, Resources and Environment/Key Laboratory of Grassland Resources of the Ministry of Education/Key Laboratory of Forage Cultivation, Processing and High Utilization of the Ministry of Agriculture/Key Laboratory of Grassland Management and Utilization of Inner Mongolia Autonomous Region Inner Mongolia Agricultural University Hohhot China

**Keywords:** geostatistics, small scale, spatial aggregation, spatial distribution, temperate grassland

## Abstract

Understanding how grazing activity drives plant community structure or the distribution of specific species in a community remains a major challenge in community ecology. The patchiness or spatial aggregation of specific species can be quantified by analyzing their relative coordinates in the community. Using variance and geostatistical analysis methods, we examined the quantitative characteristics and spatial distribution of *Stipa breviflora* in a desert steppe in northern China under four different grazing intensities (no grazing, NG, light grazing, LG, moderate grazing, MG, and heavy grazing, HG) at three small spatial scales (10 × 10 cm, 20 × 20 cm, 25 × 25 cm). We found that grazing significantly increased cover, density, and proportion in standing crop of *S*. *breviflora*, but decreased height. The spatial distribution of *S. breviflora* was strongly dependent upon the sampling unit and grazing intensity. The patchiness of *S. breviflora* reduced with sampling scale, and spatial distribution of *S. breviflora* was mainly determined by structural factors. The intact clusters of *S. breviflora* were more fragmented with increasing grazing intensity and offspring clusters spread out from the center of the parent plant. These findings suggest that spatial aggregation can enhance the ability of *S. breviflora* to tolerate grazing and that smaller isolated clusters are beneficial to the survival of this dominant species under heavy grazing.

## 1 INTRODUCTION

Grazing is a crucial regulator of ecosystem processes in grasslands (Teague et al., 2011). Grazing results in effects at different temporal and spatial levels of organization, from individual to ecosystem (Brown & Allen, 1989). Abundant studies suggest that grazing by herbivores impacts plant diversity (Sala et al., 2000), primary production (Sasaki, Okayasu, Jamsran, & Takeuchi, 2008), plant community composition (Knapp et al., 1999), as well as spatial patterns of population distribution. For example, Altesor et al. (2006) studied the responses of vegetation structure, soil attributes, and mesofauna to herbivore grazing at community and ecosystem levels and found that grazing had limited the increase of shrub cover and had redistributed soil carbon in the profile. Wan, Bai, Schönbach, Gierus, and Taube (2011) tested the responses of aboveground biomass to two grazing management systems across different levels of organization (i.e., species, plant functional group, community), and found that under continuous grazing or haymaking aboveground biomass production at all organizational levels was reduced, whereas annual alternation of grazing and haymaking had no pronounced effects on aboveground biomass.

Many studies have also examined the impact of grazing on population spatial patterns. Grazing may affect individual plants and plant populations through several mechanisms, such as removal of plant shoot issues, dung and urine return, and trampling (Chen, Christensen, Nan, & Hou, 2017). Grazing alters life history strategy and resource utilization responses of individuals and plant populations, alters competition for limited resources and changes adaptive strategies, resulting in different spatial distributions of plant population or communities. In general, spatial distribution and population dynamics reflect the impacts of environment on individuals’ survival and population growth as well as the responses through ecological adaptive strategies of indicator plants (Dieckmann, Herben, & Law, 1997). Spatial scale is an important dimension to assess when investigating mechanisms underpinning the observed changes in a population (Bai et al., 2012), and spatial distribution is strongly dependent on spatial scale. Few studies, however, have examined the mechanisms of spatial distribution of plant individual or population responses to grazing at different small scales (Kleijn & Steinger, 2002).

**Figure 1:**
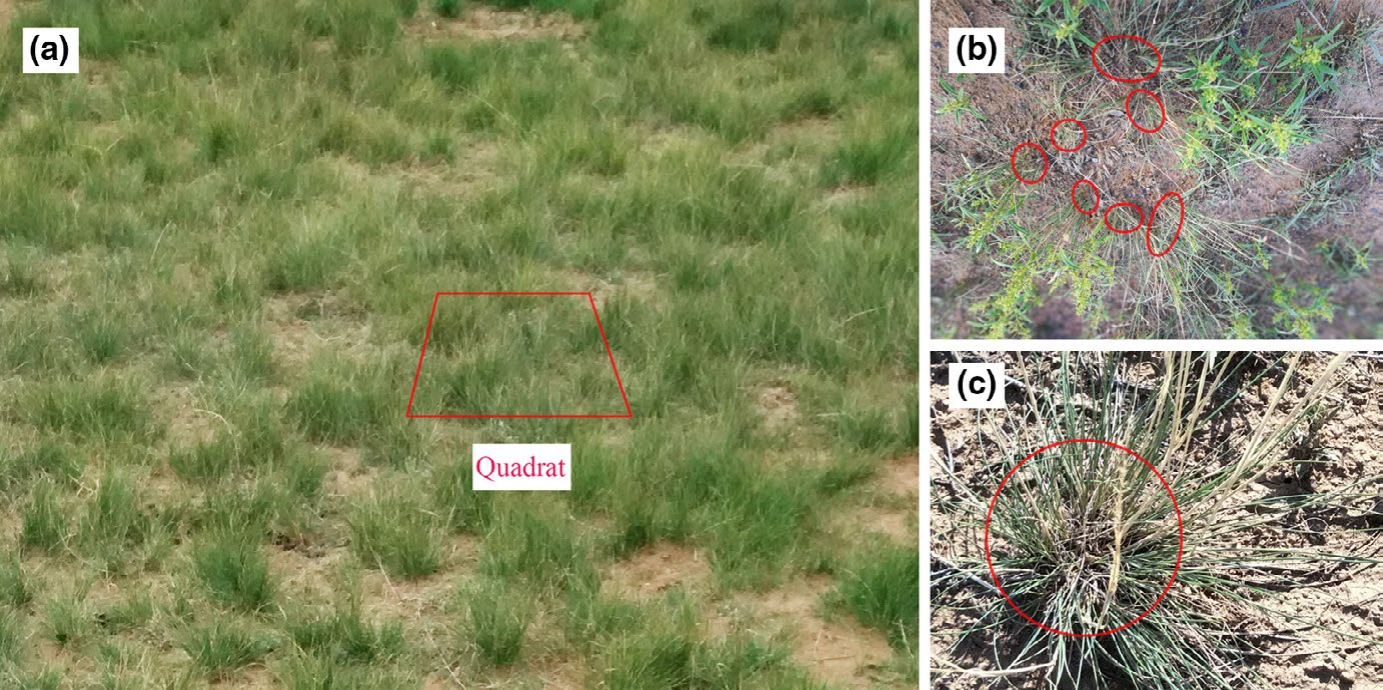
*Stipa breviflora* in the desert steppe. (a) Sampling quadrat (4 m^2^); (b) Divided into several smaller isolated clusters; (c) Intact cluster. The square indicates the sampling quadrat, and the circles indicate clusters of *S. breviflora*

Strategies to cope with grazing by herbivores differ among plant species, and spatial aggregation—in which an intact plant cluster divides into several smaller isolated clusters (Figure 1)—can enhance the tolerance of clone plants to stresses (Wang et al., 2017). There are two forms of clonal growth: phalanx and guerrilla (Lovett‐Doust, 1981). Liu, Yu, Ye, and Dong (2007) suggest that *Cleistogenes squarrosa*, a phalanx clonal grass, has an advantage in growing ramets under stressful and low nutrient conditions. Wang et al. (2017) suggest that *Iris delavayi*, another phalanx clonal grass, can maintain productivity through clonal integration with intact ramets under heavy defoliation.

A number of previous experiments have used defoliation to simulate foraging under different levels of grazing intensity. Yet few field experiments used grazing by domestic livestock (e.g., sheep) to test the response of spatial aggregation to external stresses. *Stipa breviflora* is a phalanx clonal plant and a dominant species in the desert steppe of northern China. Our study examined the effects of a grazing gradient on the spatial distribution of *S. breviflora* at different small spatial scales. Using geostatistical analysis, this paper addressed the following questions: (a) Does *S. breviflora* density increase along the grazing intensity (from 0 stocking rate to 0.91, 1.82, and 2.71 sheep·hm^−2^·half year^−1^)? And if so, (b) what kind of spatial aggregation process can be identified at different scales?

## 2 MATERIALS AND METHODS

### 2.1 Study site

The experimental region (41°46′43.6″N, 111°53′41.7″E; elevation 1,456 m) is a desert steppe in Siziwang Banner in Inner Mongolia, northern China. The region has a temperate continental arid and semiarid climate, characterized by significant inter‐ and intra‐annual variability of hydrothermal conditions.

Topography is mainly low rolling hills. Mean annual precipitation is 223 mm and mean annual temperature is 6.31°C, ranging from –17.61°C in January to 21.12°C in July (from 2004 to 2016). The main soil type is light chestnut soil (Chinese classification) or calcic kastanozems (FAO soil classification),and soil texture is sandy loam with 71.23 ± 3.98% sand, 15.97 ± 2.87% silt, and 12.81 ± 2.69% clay (Ding et al., 2016). The vegetation in this area is dominated by *Stipa breviflora*, *Artemisia frigida*, and *Cleistogenes songorica*, with average vegetation height of 5 cm and a canopy cover ranging from 17% to 20%.

### 2.2 Experimental design

**Figure 2:**
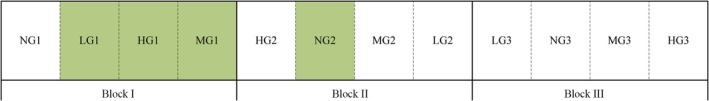
Schematic diagram for the grazing experiment plots. Dark color plots indicate sampling plots. The grazing experiment plots (each ca. 4.4 ha) were arranged in a randomized complete block design, which included four stocking rate treatments with three repeats at each stocking rate. The stocking rates were 0, 0.91, 1.82, 2.71 sheep·hm^−2^·half year^−1^ representing no grazing (NG), light grazing (LG), moderate grazing (MG), and heavy grazing (HG), respectively

To quantitatively test the effects of grazing intensity on the desert steppe ecosystem, twelve adjacent plots (each ca. 4.4 ha) were established at a grazing experimental site in 2004. The plots were arranged in a randomized complete block design, which included four stocking rate treatments with three repeats for each stocking rate (Figure 2). Seasonal grazing started on 1 June and ended on 31 November in each year since 2004. The initial sheep from the same cohort were 2‐year‐old Mongolian wethers and individuals were replaced after 3 years. The daily grazing schedule was from 6:00 a.m. to 6:00 p.m. Water and salt were provided (Wang et al., 2011).

### 2.3 Vegetation sampling

In August 2016, for sampling of herbaceous species, ten 50 cm × 50 cm quadrats were randomly positioned to record species name, height, density, and cover in each plot. As shown in Figure 1, density was counted by either isolated clusters (Figure 1b) or intact clusters (Figure 1c). Then, the standing biomass was clipped at the soil surface, oven‐dried at 65°C, and weighed. Four plots were selected to represent the LG, MG, HG plots in block I and the NG plot was selected in block II in order to avoid edge effects because the NG plot in block I is located at the edge of the experimental site (Figure 2). In each of the four plots, one 2 × 2 m quadrat was selected. In each quadrat, a 1 × 1 m quadrat frame with 10 × 10 cm grids was placed four times from left to right and from top to bottom of the quadrat sequentially, and a tape measure used to identify the precise spatial locations of *S. breviflora* in the quadrat. The origin of the coordinates was defined as the upper left corner of the quadrat.

### 2.4 Data analysis

The basic quantitative characteristics of *S. breviflora* in the NG, LG, MG, and HG treatments were indicated by cover (%), density (clusters/m^2^), height (cm), and proportion of standing crop in the community (%). Since the data on cover, density, height, and proportion of standing crop in the community did not conform to the normal distribution, square root transformation was carried out for cover, density, and height, and extreme values of cover were removed, so that the cover, density, and height data conformed to normality. Since the proportion of standing crop is limited to the range 0–1, the arcsine transformation was carried out to transform the data to a normal distribution. A generalized linear model (GLM) was used to test the effects of grazing intensity on the basic quantitative characteristics of *S. breviflora* in the treatments, and we used Duncan's test (Levene's test for homogeneity) to compare grand means among the grazing treatments. Then, subquadrats of 10 × 10 cm, 20 × 20 cm, and 25 × 25 cm sampling scales were defined by dividing the 2 × 2 m quadrat into 400, 100, and 64 subquadrats, respectively. 10 × 10 cm was the size of the majority of intact clusters of *S. breviflora* in the NG treatment (as measured by the authors), while 20 cm × 20 cm was double the minimum scale and 25 × 25 cm was defined considering sample size. The design of the spatial scale gradient can reflect whether the density of *S. breviflora* increased with increasing scale and whether the scale of spatial autocorrelation increased. The density of *S. breviflora* was calculated in each subquadrat. Two‐way GLM was used to test the effects of grazing intensity and spatial scale on the density of *S. breviflora* in the plots and generalized linear model was used to test the effects of spatial scale on the density of *S. breviflora* in the plots. Variance analysis was undertaken using SAS 9.4 (SAS Institute Inc.) at the *p* < 0.05 level of significance.

Before geostatistical semivariogram analysis, the skewness (*S*), kurtosis (*K*), and confidence intervals of the sample data distribution were calculated. If all skewness and kurtosis were contained within the intervals, the sample data were considered to have a normal distribution.

The calculations were performed in Excel 2010 (Microsoft Inc.), and the sample data fitted within the skewness and kurtosis confidence intervals. The density of *S. breviflora* in each square (10 × 10 cm, 20 × 20 cm, and 25 × 25 cm) was analyzed by geostatistics. Using a kriging method for spatial interpolation, the pattern maps of *S. breviflora* were graphed according to the semivariogram (Matheron, 1963):

$$r(h)=\frac{1}{2N(h)}\sum_{x_{i}=1}^{n}{\left[Z(x_{i}+h)\right]}^{2}$$

where *r(h)* is the semivariogram when the sample spacing is *h*; *N(h)* refers to the number of samples with interval *h*; and *Z(x_i_)* and *Z(x_i_ + h)* represent the measured values in the corresponding locations, respectively.

**Figure 3:**
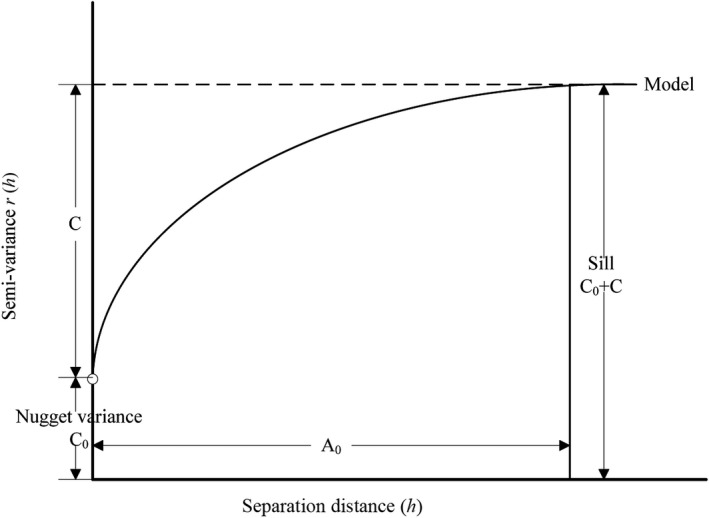
Semivariogram model

In order to quantitatively study the spatial autocorrelation of *S. breviflora* density, spatial interpolation optimal theoretical models (linear, exponential, Gaussian, or spherical model, Figure 3) were used for semivariance optimum fitting. Analysis was performed using GS^+^9.0 (Gamma Design Software, LLC). The number of lags was 1, which stands for the 10, 20, and 25 cm interval scales, respectively, and is related to the coordinates of the response scale. The lag interval used was the default setting and was generally equal to 1/2 of the maximum sample scale. Spatial autocorrelation is used to reflect spatial heterogeneity. Thus, the stronger the spatial autocorrelation, the lower the heterogeneity, the higher the uniformity, and the higher the degree of spatial aggregation. The optimal fit model was used to determine the preliminary type of curve according to the scatter diagram (Clark, 1981), and then the principle of least squares was used to estimate the parameters of the initial type curve and to determine the optimal curve (Cressie, 1991). In this model, Nugget Variance (*C*_0_), Sill (*C*_0_ + *C*), Structural ratio (*C*/(*C*_0_ + *C*)), and Range parameter (*A*_0_) are important parameters (Table 1, Figure 3). When *C*/(*C*_0_ + *C*) is less than 25%, this represents weak spatial autocorrelation, while between 25% and 75% represents moderate spatial autocorrelation, and greater than 75% represents strong spatial autocorrelation. A (Range) indicates the maximum distance of spatial correlation. Sometimes this is called the effective range in order to distinguish the range (*A*) from a model's range parameter (*A*_0_). In GS+, the Range (*A*) is calculated from *A*_0_ as described in the formulae for the different models, where the range of spatial autocorrelation of the linear, exponential, and spherical models was *A*_0_, 3 *A*_0,_ and *A*_0_, respectively.

**Table 1:** Semivariogram parameters

Parameters	Abbreviation	Interpreting
Nugget or Nugget variance	*C*_0_	The *y*‐intercept of the model; spatial variation caused by random factors.
Sagitta	*C*	Spatial variation caused by structural factors (e.g., soil, topography, physiognomy, etc.).
Sill	*C*_0_ + *C*	The model asymptote;
Structural ratio	*C*/(*C*_0_ + *C*)	The proportion of structural spatial distribution factors in the maximum spatial variation.
Range parameter	*A*_0_	The model's parameter is used to calculate effective range.
Effective range or Range	*A*	The maximum distance of spatial correlation.
Determination coefficient	*r* ^2^	Testing the optimal fitting model; the larger, the better
Residual sum of squares	RSS	Testing the optimal fitting model; the smaller, the better

## 3 RESULTS

### 3.1 The quantitative characteristics of *S. breviflora*

**Figure 4:**
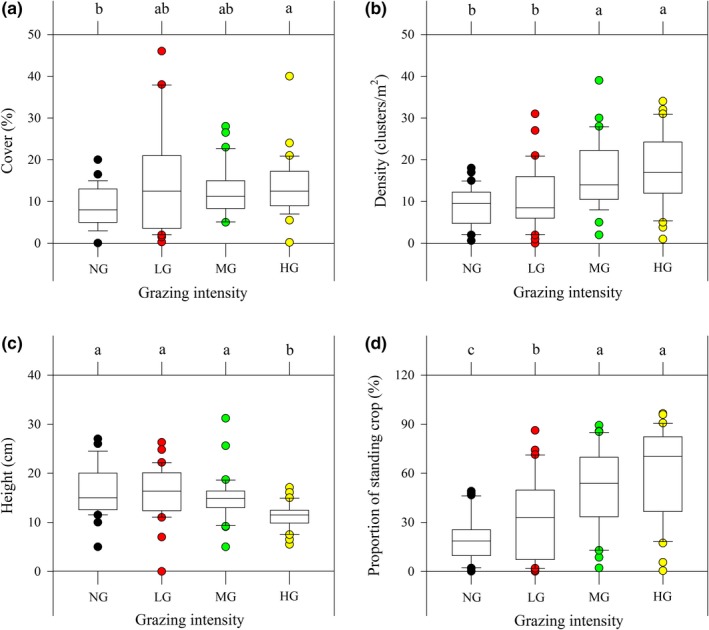
Effects of grazing on the basic quantitative characteristics of *Stipa breviflora* in the grazing treatments. Mean values (±*SD*; *n* = 30) of (a) cover, (b) density, (c) height and (d) proportion of standing crop. Different lowercase letters indicate that grand means differ significantly between the grazing treatments (*p* < 0.05)

Grazing intensity significantly affected the basic quantitative characteristics of *S. breviflora* (Table 2). Cover of *S. breviflora* was significantly lower under NG than under LG and HG, but did not differ between NG and MG or between LG and HG (Figure 4a). Density of *S. breviflora* was significantly higher under MG and HG, but density did not differ between NG and LG or between MG and HG (Figure 4b). Grazing significantly decreased the height of *S. breviflora*, but only under HG (Figure 4c). The proportion of standing crop of *S. breviflora* increased markedly with increasing grazing intensity (Figure 4d).

**Table 2:** The effects of interaction between grazing and sampling scale on *Stipa breviflora* density

	Density		
Impact factors	*df*	*F* value	*P* value
Stocking rate	3	45.70	<0.001
Scale	2	505.75	<0.001
Stocking rate × Scale	6	9.35	<0.001

### 3.2 Change in the density of *S. breviflora* population at different scales

**Figure 5:**
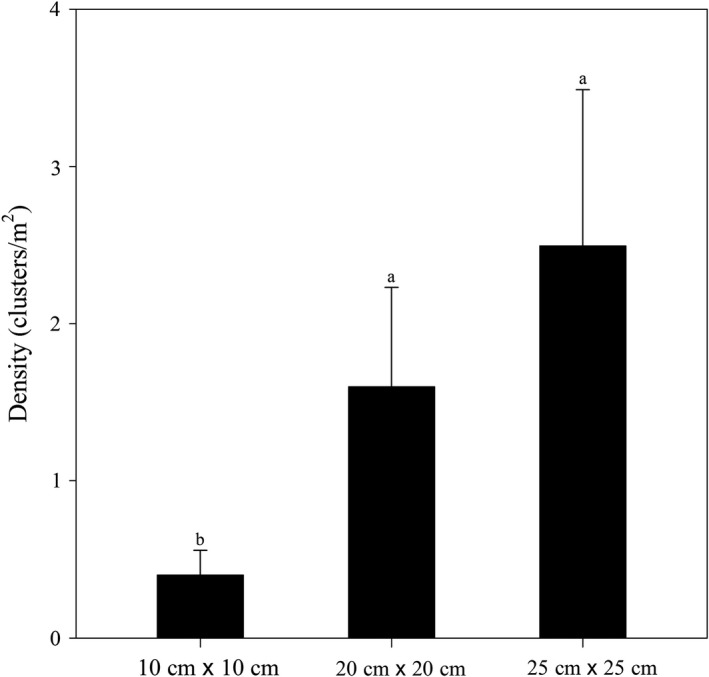
Effects of sampling unit on density of *Stipa breviflora*. Mean values ± *SD* (*n* = 4). Different lowercase letters indicate that grand means differ significantly between the spatial scales (*p* < 0.05)

Spatial scale significantly affected the density of *S. breviflora* (Table 2). Density increased markedly with increasing spatial scale, and it dramatically increased by approximately 300% as the spatial scale shifted from 10 × 10 cm to 20 × 20 cm, while density increased by approximately 56% from 20 × 20 cm to 25 × 25 cm (Figure 5). The increase in the range of density from 10 × 10 cm to 20 × 20 cm is relatively large, while the increase from 20 × 20 cm to 25 × 25 cm is smaller.

### 3.3 The effects of grazing intensity on *S. breviflora* spatial heterogeneity

**Figure 6:**
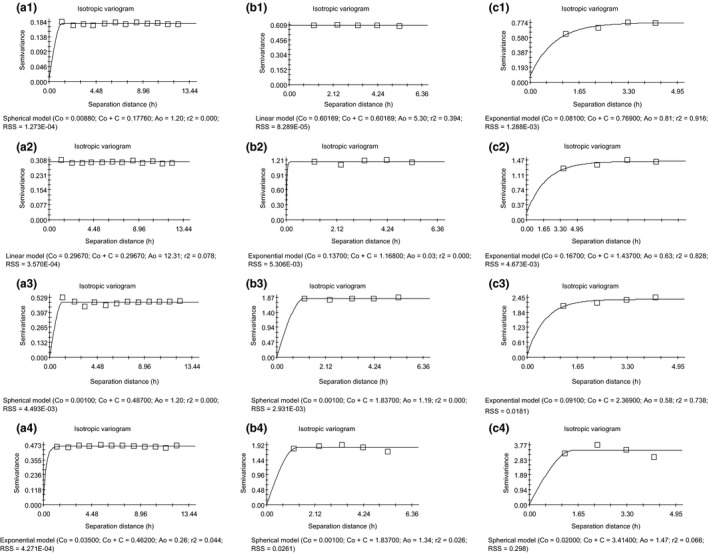
The semivariogram of *Stipa breviflora* spatial distribution under different grazing intensities. (a) 10 × 10 cm; (b) 20 × 20 cm; and (c) 25 × 25 cm. 1, No grazing; 2, light grazing; 3, moderate grazing; 4, heavy grazing

In the NG treatment, spherical, linear, and exponential models were best fitted at 10 × 10 cm, 20 × 20 cm, and 25 × 25 cm scales, respectively (Table 3). We found the largest spatial variation was caused by random factors (*C*_0_ = 0.602, Nugget, also called nugget variance) at the 20 × 20 cm scale (Figure 6b1), and the largest spatial variation was caused by structural factors (*C* = 0.688, structural variance) at the 25 × 25 cm scale (Figure 6c1). The maximum spatial variation (*C*_0_ + *C*) at the 25 × 25 cm scale was 0.769. The largest structure ratio (*C*/(*C*_0_ + *C*) = 99.95%) appeared at the 10 × 10 cm scale. The range of spatial autocorrelation of *S. breviflora* is 12, 160, and 60.75 cm at 10 × 10 cm, 20 × 20 cm, and 25 × 25 cm scales, respectively. Thus, the range of spatial autocorrelation of *S. breviflora* differed at different scales. Combining the structural ratio of the semivariogram, we found that the range of spatial autocorrelation is the largest when structural ratio is the smallest, and vice versa. Combining the explanation of spatial variation and the spatial autocorrelation of *S. breviflora*, we found that the range of spatial autocorrelation is controlled by structural factors at 10 × 10 cm and 25 × 25 cm scales and by random factors at 20 × 20 cm scale.

**Table 3:** The relevant measures of curve‐fitted semivariograms at different scales under different grazing intensity treatments

		Optimal model and parameters^a^						
Scale	Stocking rate	Model	*C*_0_	*C*_0_ + *C*	*C*/(*C*_0_ + *C*) (%)	*A*_0_	*r* ^2^	RSS
10 × 10‐cm (A)	NG (1)	Spherical	0.009	0.178	94.94	1.20	0.000	1.273 × 10^−4^
	LG (2)	Linear	0.297	0.297	0.00	12.31	0.078	3.570 × 10^−4^
	MG (3)	Spherical	0.001	0.487	99.79	1.20	0.000	4.493 × 10^−3^
	HG (4)	Exponential	0.035	0.462	92.42	0.26	0.044	4.271 × 10^−4^
20 × 20‐cm (B)	NG (1)	Linear	0.602	0.602	0.00	5.30	0.394	8.289 × 10^−5^
	LG (2)	Exponential	0.137	1.168	88.27	0.03	0.000	5.306 × 10^−3^
	MG (3)	Spherical	0.001	1.837	99.95	1.19	0.000	2.931 × 10^−3^
	HG (4)	Spherical	0.001	1.837	99.95	1.34	0.026	4.271 × 10^−4^
25 × 25‐cm (C)	NG (1)	Exponential	0.081	0.769	89.47	0.81	0.916	1.288 × 10^−3^
	LG (2)	Exponential	0.167	1.437	88.38	0.63	0.828	4.673 × 10^−3^
	MG (3)	Exponential	0.091	2.369	96.16	0.58	0.738	0.0181
	HG (4)	Spherical	0.020	3.414	99.41	1.47	0.066	0.2980

Abbreviations: HG, heavy grazing; LG, light grazing; MG, moderate grazing; NG, no grazing.

a For parameter meanings see Table 1.

In the LG treatment, the best fitted models of the semivariogram were linear and exponential models (Table 3). The largest spatial variation caused by random factors was 0.297 at the 10 × 10 cm scale (Figure 6a2). The largest spatial variation caused by structural factors was 1.270 at the 25 × 25 cm scale (Figure 6c2). The maximum spatial variation (*C*_0_ + *C*) was 1.437 at the 25 × 25 cm scale, and the largest structure ratio (*C*/(*C*_0_ + *C*)) was 88.38% at 25 × 25 cm scale. Therefore, the spatial variation caused by random factors (which had the smallest value at 20 × 20 cm scale) had no relevant relationship with scale division. Spatial variation was mainly caused by structural factors, and the largest spatial variance and the structure ratio increased with spatial scale. The range of spatial autocorrelation of *S. breviflora* was 123.1, 18, and 15.75 cm at three spatial scales (10 × 10 cm, 20 × 20 cm, and 25 × 25 cm), respectively. Thus, the range of spatial autocorrelation of *S. breviflora* decreased with increasing spatial scale, suggesting that the heterogeneity of *S. breviflora* increased with spatial scale.

In the MG treatment, the best fitted models of the semivariogram were spherical and exponential models (Table 3). The largest spatial variation caused by random factors and structural factors both occurred at 25 × 25 cm scale (Figure 6c3), the variation of which were 0.091 and 2.278, respectively. The largest spatial variation was 2.369 at 25 × 25 cm scale, and the largest structure ratio was 99.99% at 20 × 20 cm scale. Random factors, structural factors, and spatial variation increased with spatial scale. The range of spatial autocorrelation of *S. breviflora* was 12 and 23.8 cm, and 43.5 cm at 10 × 10 cm, 20 × 20 cm, and 25 × 25 cm scale, respectively. Thus, the range of spatial autocorrelation of *S. breviflora* increased with increasing spatial scale. The range of spatial autocorrelation, random factors, structural factors, and spatial variation increased with increasing scale, which indicates that the heterogeneity of *S. breviflora* reduced with increasing scale and that spatial variation was mainly affected by structural factors.

In the HG treatment, the best fitted models of the semivariogram were spherical and exponential models (Table 3). The largest spatial variation caused by random factors was 0.035 at 10 × 10 cm scale (Figure 6a4), and the largest spatial variation caused by structural factors was 3.394 at 25 × 25 cm scale (Figure 6c4). The maximum spatial variation was 3.414 at 25 × 25 cm scale, and the largest structure ratio was 99.99% at 20 × 20 cm scale. Structural factors increased with increasing scale. The range of spatial autocorrelation of *S. breviflora* was 7.8, 26.8, and 36.75 cm at 10 × 10 cm, 20 × 20 cm, and 25 × 25 cm scale, respectively. Thus, the range of spatial autocorrelation of *S. breviflora* increased with spatial scale. Combined with the structural ratio of the semivariogram, we found that the range of spatial autocorrelation and structural factors increased with increasing scale, which indicates that the heterogeneity of *S. breviflora* decreased with spatial scale and that patterns were mainly caused by structural factors.

### 3.4 The spatial distribution of *S. breviflora* population under grazing

**Figure 7:**
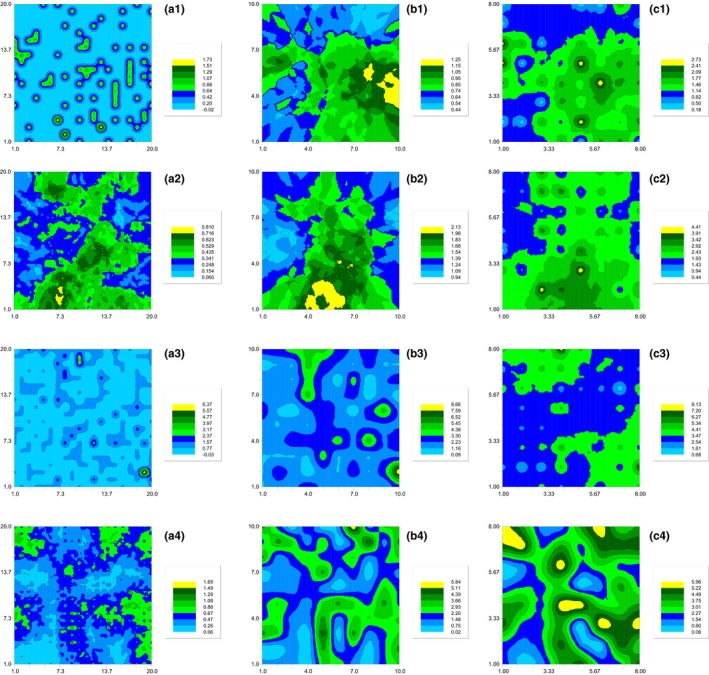
The 2‐d spatial pattern map of *Stipa breviflora* under different grazing intensities. (a) 10 × 10 cm; (b) 20 × 20 cm; and (c) 25 × 25 cm. 1, No grazing; 2, light grazing; 3, moderate grazing; 4, heavy grazing. Different color bands represent the range of different interpolations, and the greater number represents the greater population density

The 2‐d spatial pattern map directly presents the heterogeneity and complexity of *S. breviflora* spatial distribution and shows the patchiness, hierarchy, and the mosaic distribution of *S. breviflora*. At 10 × 10 cm scale, *S. breviflora* individuals are separated and independent (Figure 7a1) from each other, especially in the no grazing treatment. As spatial scale increased, species patches were gradually formed by individuals of high density, representing a large amount of new generation and indicating that regeneration of *S. breviflora* spread out from the center of the stock plant. The patchiness of spatial distribution of *S. breviflora* decreased with increasing spatial scale, whereas patch size and spatial aggregation among patches increased (Figure 7). In the LG treatment, although band distribution disappeared with increasing scale, the pattern of gathering around the stock plants remained unchanged, and spatial distribution showed randomly extensive patches at 10 × 10 cm and 20 × 20 cm scales.

However, banded distribution appeared in the HG treatment and the patchiness of the distribution reduced at 25 × 25 cm scale (Figure 7c4). We found that spatial aggregation of *S. breviflora* increased with increasing grazing intensity at all three scales. In addition, the spatial distribution was mostly fragmented under HG (Figure 7a4, b4, c4).

## 4 DISCUSSION

### 4.1 Response of *S. breviflora* population to grazing intensity

Plants have different response strategies to defoliation by herbivores. We found that grazing decreased the height of *S. breviflora*, but increased its cover, density, and proportion in the standing crop. These results suggest that *S. breviflora* has a relatively strong ability to survive grazing. With increased grazing intensity, the height of *S. breviflora* was reduced. However, the proportion in the standing crop was increased. This suggests that domestic animals prefer to search and select palatable species though selective foraging (Jamieson & Hodgson, 1979). In this study, we measured the effects of long‐term grazing across a gradient of grazing intensity on the *S. breviflora* community. We found that density increased with grazing intensity. Long‐term overgrazing (e.g., HG) leads to a decrease in the palatability of plant species, thus increasing livestock selective foraging time and frequency of trampling. Frequent trampling results in intact clusters dividing into several smaller clusters, thereby increasing the density of *S. breviflora*. The reason is that *S. breviflora* is a perennial dense cluster grass, and within one growing season every basic tillering node of the mother culm usually produces a tiller. Tillers can also undertake tillering that produces secondary tillers, finally forming tillering clusters in an exponential progression. In addition, the growing point of *S. breviflora* is above ground, and it is therefore sensitive to livestock trampling (Branson, 1953), which promotes spatial aggregation.

Furthermore, the phalanx structure of *S. breviflora* forms an effective barrier for fixing and accumulating sand (Liu, Lv, Wang, Yan, & Wei, 2018), thereby forming phytogenic hillocks that further prevent land erosion and induce sand deposition (Wang, Wang, Dong, Liu, & Qian, 2006). When a cluster of *S. breviflora* is buried by sand, the tiller node undergoes displacement (Chen, Zhang, Wang, Zhan, & Zhao, 2001). This is another reason why intact clusters of *S. breviflora* break into several smaller isolated clusters.

Compensatory growth of plants may well explain the response of *S. breviflora* to defoliation by grazing. Compensatory growth associated with grazing may result from the stimulation of photosynthesis in remaining green tissues (Anten & Ackerly, 2001), reallocation of resources (Zhao, Chen, & Lin, 2008), and/or activation of additional meristems because of release of apical dominance (Liu, Yu, He, Chu, & Dong, 2009).

### 4.2 Propagation of offspring around the center of the mother plant

The 2‐d spatial pattern maps clearly show that the contour lines became closer and the value of *S. breviflora* individual density decreased gradually from the center to the periphery. These findings indicate the regeneration capacity and diffusivity into surrounding space around the center of the mother plant. At 10 × 10 cm scale, the clusters were clearly independent of each other under NG (shown by independent closed contour lines, and mean basal diameter of *S. breviflora* branching generally around 10 cm, personal observation), whereas the clusters presented larger dense patches with increasing grazing intensity. Simultaneously, the range of spatial autocorrelation of *S. breviflora* showed that spatial heterogeneity of *S. breviflora* distribution reduced with increasing scale (from 10 × 10 cm to 25 × 25 cm) under MG and HG. These results suggest that heavy grazing intensity reduced spatial heterogeneity and promoted the mother plants to spread into the surrounding area. Phalanx plants have an advantage in acquiring local resources and therefore may have a competitive advantage in a homogeneous environment with higher spatial aggregation of clusters (Saiz, Bittebiere, Benot, Jung, & Mony, 2016). The pattern of spatial aggregation may result from limited seed or clonal dispersal, environmental heterogeneity (Xue, Huang, Yu, & Bezemer, 2018) and positive herbivore‐plant feedback, which can enhance the capacity of plants to colonize different microhabitats, to tolerate resource heterogeneity, to compete, and to recover from herbivory predation (Schmid, Puttick, Burgess, & Bazzaz, 1988).

Offspring surrounding the mother plant may be advantageous because older ramets can share resources to support the development of younger ramets (Herben, 2004). Studies have shown that it is more common for resources to be shared from developmentally older to developmentally younger ramets than for resources to be shared from developmentally younger to developmentally older ramets (Song et al., 2013). For example, Alpert (1996) reported that nitrogen moves mainly from the older to the younger ramets. Thus, it appears that physiological integration in many plants primarily provides support to the establishment of daughter ramets. Another advantage of offspring developing close to the mother plant is that physiological integration allows support of clone parts growing in low‐resource patches (Wang et al., 2009). This may represent a trade‐off with the spatial heterogeneity of resources.

## 5 CONCLUSION

We conclude that *S. breviflora* populations generate spatial aggregation with increasing grazing intensity and that offspring clusters are spread out to the surrounding area from the center of the mother plant. Our results suggest that spatial aggregation can enhance the ability of *S. breviflora* to tolerate grazing and that smaller isolated clusters are beneficial to the survival of this dominant species under heavy grazing. This may further affect ecosystem stability and sustainability in these grasslands.

## CONFLICT OF INTEREST

The authors declare no conflicts of interest.

## AUTHOR CONTRIBUTIONS

Guodong Han conceived and designed the experiments. Baolong Yan wrote the main manuscript text and performed the experiments. Shijie Lv and Zhongwu Wang processed and analyzed the data. Sarula Kang contributed in manuscript editing. All authors reviewed the manuscript.

## Data Availability

All data (cover, density, height, standing crop and relative coordinates’ data) from this manuscript are publically available in the FigShare database (https://doi.org/10.6084/m9.figshare.7326536).
